# Interventions for American Cutaneous and Mucocutaneous Leishmaniasis: A Systematic Review Update

**DOI:** 10.1371/journal.pone.0061843

**Published:** 2013-04-29

**Authors:** Ludovic Reveiz, Ana Nilce Silveira Maia-Elkhoury, Rubén Santiago Nicholls, Gustavo Adolfo Sierra Romero, Zaida E. Yadon

**Affiliations:** 1 Health Systems Based on Primary Health Care, Pan American Health Organization (PAHO), Washington, D.C., United States of America; 2 Health Surveillance Disease Prevention and Control, Communicable Diseases Project, Pan American Health Organization (PAHO), Duque de Caxias, Rio de Janeiro, Brazil; 3 Center for Tropical Medicine, University of Brasilia and National Institute for Science and Technology for Health Technology Assessment (IATS/CNPq), Porto Alegre, Rio Grande do Sul, Brazil; Royal Tropical Institute, The Netherlands

## Abstract

**Introduction:**

Leishmaniasis is an important public health problem in the Americas. A Cochrane review published in 2009 analyzed 38 randomized controlled trials (RCT). We conducted a systematic review to evaluate the effects of therapeutic interventions for American cutaneous and mucocutaneous leishmaniasis.

**Methods:**

All studies were extracted from PubMed, Embase, Lilacs (2009 to July, 2012 respectively), the Cochrane Central Register of Controlled Trials (6-2012) and references of identified publications. RCTs’ risk of bias was assessed.

**Results:**

We identified 1865 references of interest; we finally included 10 new RCTs. The risk of bias scored low or unclear for most domains. Miltefosine was not significantly different from meglumine antimoniate in the complete cure rate at 6 months (4 RCT; 584 participants; ITT; RR: 1.12; 95%CI: 0.85 to 1.47; I2 78%). However a significant difference in the rate of complete cure favoring miltefosine at 6 months was found in L. panamensis and L. guyanensis (2 RCTs, 206 participants; ITT; RR: 1.22; 95%CI: 1.02 to 1.46; I2 0%). One RCT found that meglumine antimoniate was superior to pentamidine in the rate of complete cure for L. braziliensis (80 participants, ITT; RR: 2.21; 95%CI: 1.41 to 3.49), while another RCT assessing L. guyanensis did not find any significant difference. Although meta-analysis of three studies found a significant difference in the rate of complete cure at 3 months favoring imiquimod versus placebo (134 participants; ITT; RR: 1.45; 95%CI: 1.12 to 1.88; I2 0%), no significant differences were found at 6 and 12 months. Thermotherapy and nitric oxide were not superior to meglumine antimoniate.

**Conclusion:**

Therapeutic interventions for American cutaneous and mucocutaneous leishmaniasis are varied and should be decided according to the context. Since mucosal disease is the more neglected form of leishmaniasis a multicentric trial should be urgently considered.

## Introduction

Leishmaniasis is an important public health problem in 98 endemic countries of the world, with more than 350 million people at risk. WHO estimated an incidence of 2 million new cases per year (0.5 million of visceral leishmaniasis (VL) and l.5 million of cutaneous leishmaniasis (CL). VL causes more than 50, 000 deaths annually, a rate surpassed among parasitic diseases only by malaria, and 2, 357, 000 disability-adjusted life years lost, placing leishmaniasis ninth in a global analysis of infectious diseases. In addition, most patients have very poor access to the health system resulting in important underreporting of cases [1]–[6].

In the Americas, leishmaniases endemic areas extend from Mexico to Argentina. Approximately 67,000 clinical cases are reported every year and 40,840,000 people are at risk of developing the disease in over 21 countries, with estimated of 187,200 to 307,800 CL cases [1], [6], [7], and 4,500 to 6,800 VL cases [6]. While more than 90% of the VL cases occur in India, Bangladesh, Sudan, Ethiopia and Brazil, approximately 70% of CL cases occur in Afghanistan, Algeria, Colombia, Brazil, Iran, Syria, Sudan, Ethiopia, Nicaragua and Peru [5], [6].

The leishmaniases are diseases caused by different species of parasites of the genus *Leishmania* and transmitted by vectors family Psychodidae infected from different reservoirs; it is also characterized by a large clinical polymorphism. Fifteen *Leishmania* species were identified as pathogenic to humans being causing cutaneous, mucosal (ML) and visceral leishmaniasis. The cutaneous and mucosal forms have a broad clinical spectrum that range from single or multiple localized skin lesions to severe diffuse and mucosal lesions [5], [8].

The epidemiology of the leishmaniases is dynamic and the circumstances of transmission are continually changing in relation to environment, demography, human behavior, socioeconomic status, and other factors such as immunogenic profile of affected human populations [9]–[15].

In the New World, cutaneous leishmaniasis is caused by a variety of species belonging to the subgenera *Leishmania* and *Viannia* producing different clinical manifestations; however, part of the population have subclinical infections. Although the most frequent clinical form of cutaneous leishmaniasis presents as single or multiple lesions, disseminated lesions can also be observed. The lesions may occur anywhere in the body but commonly originate at the site of inoculation where initially a macular lesion forms, followed by a papule and then by a nodule that progressively increases in size and becomes ulcerated. These lesions can develop in weeks, months or years after infection [5], [10], [16].

Although lesions caused by *L. mexicana* may heal spontaneously in an average period of 4 months, this species and other such as *L. amazonensis, L. venezuelensis*, and *L. pifanoi* can cause diffuse cutaneous leishmaniasis, considered an anergic, severe, and chronic form of the disease. The response to the first therapeutic scheme is frequently unsatisfactory, due to changes in immunological conditions, physiological or nutritional characteristics of patients or to specific pharmacokinetics factors of drugs used [5], [17]–[19].

An atypical form of CL has been described at the same geographical area of VL presenting circumscribed and non- ulcerated lesions; it mainly affects older children and young adults, while visceral leishmaniasis presents predominantly in children less than 5 years. This clinical form is caused by *L. infantum (*syn. *L. chagasi)* that can evolve into a visceral form, in patients with deprived immunological conditions [20], [21].

Some species of the subgenus *Viannia* such as *L. braziliensis*, *L. panamensis,* and *L. guyanensis* might disseminate (metastasis) from the primary lesion to a distant mucosal site, leading to destructive secondary lesions especially in the nasopharyngeal areas. More rarely the musosal lesion might result by contiguity, for instance, skin lesion near the nasal or oral mucosa. This form does not evolve spontaneously to clinical cure, and if left untreated, develops to mutilation or destruction, affecting the quality of life of patients. In general, treatment failures and relapses are common in this clinical form [18], [22], [23].

In recent years, the relative proportion of mucosal leishmaniasis cases reported in the Americas is 3.1% among all the cutaneous leishmaniasis cases, however, depending on the species involved, genetic and immunological aspects of the hosts as well as the availability of diagnosis and treatment, in some countries that percentage is more than 5% as occurs in Bolivia (12–14.5%), Peru (5.3%), Ecuador (6.9–7.7%) and Brazil (5.7%) [24]–[27].

The diagnosis of CL is based on a combination of the epidemiological history (exposure), the clinical signs, symptoms, and the laboratory diagnosis which can be done either by the observation of amastigotes on Giemsa stained direct smears from the lesion or by histopathological examination of a skin biopsy. However, the sensitivity of the direct smear varies according to the duration of the lesion (sensitivity decreases as the duration of the lesion increases). Cultures and detection of parasite DNA through the polymerase chain reaction (PCR) can also be done but they are costly and their use is limited to reference or research centers. The diagnosis of mucosal leishmaniasis is based on the presence of a scar of a previous cutaneous lesion, which might have occurred several years before, and on the signs and symptoms. A positive Montenegro Skin Test (MST) and/or positive serological tests such as the immunofluorescent antibody test (IFAT) allow for indirect confirmation of diagnosis. Parasitological confirmation of mucosal leishmaniasis is difficult because the parasites are scarce and rarely found in tissue samples. Thus, histopathology not only is invasive but also demonstrates low sensitivity. This has led to the development of PCR techniques [28] which, though sensitive and specific, are still limited to research and reference laboratories.

Although pentavalent antimonial drugs are the most prescribed treatment for CL and ML, diverse other interventions have been used with varying success [29]. These include parenteral treatments with drugs such as pentamidine, amphotericin B, aminosidine and pentoxifylline, oral treatments with miltefosine, and topical treatments with paromomycin (aminosidine) and aminoglycosides. Other treatments such as immunotherapy and thermotherapy have also been tested.

The limited number of drugs available, the high levels of side effects of most of them, and the need of parenteral use, which may require hospitalization, and the fact that the use of local and oral treatment might increase patients’ compliance, highlight the need of reviewing the current evidence on efficacy and adverse events of the available treatments for American cutaneous and mucocutaneous leishmaniasis.

To identify and include new evidence on the topic, we decided to update the Cochrane review published in 2009, which identified and assessed 38 randomized controlled trials also found a number of ongoing trials evaluating diverse interventions such as miltefosine, thermotherapy and imiquimod [29]. The objective of this paper is to present a systematic review which evaluates the effects of therapeutic interventions for American CL and ML.

## Methods

### Literature Search

We carried out a literature search to identify studies assessing the effects of therapeutic interventions for American CL and ML. Searched were planned to update findings of the Cochrane systematic review published in 2009 [29]. Structured searches were conducted in PubMed (January 2009 to July 2012), the Cochrane Library (number 6, 2012), and LILACS (January 2009 to July 2012) using a comprehensive list of key terms that were adapted to each database (Supporting Information S1. Search strategies). We searched the International Clinical Trials Registry Platform search portal of WHO (ICTRP) to identify past and ongoing trials using the key word “leishma*. The references of both included and excluded material were examined in effort to find further relevant papers. We also completed a search in Scirus (limits: medicine, article title; July, 2012) to identify studies published in other databases. We reached out to authors and relevant key stakeholders to identify unpublished studies and related additional data from manuscripts. No language restrictions were applied.

### Study and Information Selection

The titles, abstracts, and studies identified in the literature search were assessed by two reviewers. We included randomized clinical trials (RCT) which assessed the effects of interventions for treating CL and ML. Subjects having CL and/or ML or VL by clinical presentation and confirmed by histopathology, polymerase chain reaction (PCR) analysis or culture of lesions were included. We considered any intervention compared with no intervention, placebo, or other treatment regimens. Studies in which the intervention group included vaccines were excluded. All studies matching the inclusion criteria were reviewed by the authors and disagreement on inclusion was settled through discussion.

### Data Extraction and Outcomes

At least two reviewers (ANM-E and LR) independently extracted the relevant data using a predesigned data extraction form; disagreements between reviewers were resolved by referring to a third author. Taking into account that a Cochrane review assessed and extracted data from previously published trials, we focused our assessment on updating provided evidence. Therefore, we designed a data collection form to systemically extract data from RCTs published later than previous the Cochrane review. The authors examined retrieved papers, identified, and recorded the main characteristics of the study including: qualitative aspects (such as date of publication, study design, geographical location and setting, population description, selection criteria, patient samplings, and funding source), characteristics of participants (age, sex, ethnicity, socioeconomic status), species of causative *Leishmania*, interventions (i.e. type, duration, method used to measure) and outcomes (type of outcome, outcome assessment method, type of statistical analysis, adjustment variables) and the risk of bias.

Clinical and/or parasitological cure at least three months after the end of treatment were the main outcomes considered in the review regardless of the microbiological method used to diagnose leishmaniasis. We defined cured as disappearance of all inflammatory signs (either skin edema or hardening, or both), and the occurrence of scarring or epithelialization of in ulcerative lesions [29]. We also extracted data on recurrence; the degree of functional and aesthetic impairment and/or prevention of scarring; emergence of resistance; and mortality. We also included those adverse events reported in RCTs and did not search for additional adverse event studies or records. Findings are presented according to categories that were pre-specified by the trial.

We performed an evaluation on the risk of bias for each new identified trial following the Cochrane Collaboration tool for the assessment of these variables [30]. We also extracted information on inclusion and exclusion criteria; sample size calculation; and baseline comparability of age, gender, relevant clinical characteristics, and diagnoses. We registered data in the studies’ table (Table 1). When necessary, authors were contacted to obtain additional information about their studies.

**Table 1:** Characteristics of included studies.

Reference	Methods	Participants	Interventions	Outcomes
Chrusciak-Talhari 2011 (Brazil) [73]	Open label randomized trial at a dermatology outpatient clinic	Patients having clinical diagnosis of CL; illness duration of less than 3 months; visualization of *Leishmania* amastigotes on Giemsa; no previous *Leishmania* treatment. Exclusion criteria HIV patients and pregnant women. Identification of *Leishmania Viannia* by PCR-RFLP on skin biopsies from enrolled patients. *L. guyanensis*, *L. braziliensis* and *L. lainsoni* were identified.	Miltefosine administered orally at the total target daily dosage of 2.5 mg/kg of body weight (maximum daily dose of 150 mg) for 28 consecutive days. Glucantime administered intravenously at a dose of 20 mg Sb +5/kg/day (age group 13–65 y/o) and 15 mg Sb +5/kg/day (age group 2–12 y/o) for 20 consecutive days (maximum daily dose of 3 ampoules),	Cure rate at 1,2,4,6 months; adverse events
Lopez 2012 (Colombia) [71]	Open label randomized trial at five military health clinics in Colombia	Positive parasitologic diagnosis of leishmaniasis; no previous treatment for this parasitic infection; laboratory exams including renal, hepatic and hematologic testing and; voluntary agreement to participate. Excluded: patients with chronic concomitant diseases; lesions compromising the mucosa; presence of 10 or more cutaneous lesions with a negative Montenegro test; cutaneous lesions located less than 2 cm from the nasal or oral mucosa, eyes or near the anal or urogenital orifices. Identificacion of Leishmania type was done from histologic samples using PCR-RFLP. *L. panamensis* and *L. brazililensis* were identified.	Thermotherapy: single session, active borders and peripheral area of the lesions. Each thermal application was at 50°C and lasted for 30 seconds; the number of applications depended on the size of the lesion. Fusidic acid was applied over the lesions for 10 days. Meglumine antimoniate (Glucantime®) was administered intramuscularly under medical supervision at a dose of 20 mg Sb5/kg/day for 20 days. Meglumine antimoniate was provided as rescue therapy for all patients	Cure rate at 6 months. “Complete reepithelialization of all ulcers and complete loss of induration up to three months after the end of treatment”; recurrence; reinfection; adverse events
López-Jaramillo 2010 (Colombia) [81]	Double-blind, randomized clinical trial at local hospitals in Santander and Tolima, Colombia	Inclusion criteria: >10 years of age; a parasitological diagnosis of CL with demonstration of *Leishmania* amastigotes on smears or promastigotes in culture. Exclusion criteria: any history of anti- Leishmania therapy in the last 3 months, presence of >5 lesions, or presence of lesions in the perimeter (<2 cms) of mucosal areas, eyes, nose, mouth, or genitals. CL caused by *L. panamensis*.	Meglumine antimoniate (Glucantime) 20 mg/kg/day plus a placebo patch for 20 days. Intramuscular placebo (5–20 cc/day), and an active Nitric Oxide releasing patch for 20 days.	Cure rate at 90 days; relapse; reinfection; adverse events
Machado 2010 (Brazil) [74]	Open label randomized trial at the health post of Corte de Pedra, Bahia, Brazil.	Inclusion criteria: presence of a typical ulcerated lesion and a positive Montenegro intradermal skin test in a subject living in the endemic area; age 2–65 years; a maximum of 5 ulcers with no more than 2 body regions involved; lesion size between 10 and 50 mm in a single dimension; a period of less than 90 days from the onset of the first ulcer. Punch biopsy to obtain material for *Leishmania* culture and PCR. Exclusion criteria: prior history of CL or antimony use, evidence of mucosal or disseminated disease, pregnancy or breastfeeding; HIV or any systemic severe disease. *L. (V.) braziliensis* was identified.	Miltefosine orally at the total target daily dosage of 2.5 mg/kg of body weight (maximum daily dose of 150 mg) for 28 consecutive days. Pentavalent antimony intravenously at a dose of 20 mg Sbv/kg/day for 20 consecutive days (maximum daily dose of 3 ampoules or 1215 mg/Sbv).	Cure rate at 2 weeks, 1, 2, 4 and 6 months; relapses; adverse events
Miranda-Verastegui 2009 (Peru) [76]	Randomized double-blind clinical trial. at the Instituto de Medicina Tropical ‘Alexander von Humbolt’–Hospital Nacional Cayetano Heredia in Lima and Cusco, Peru	Inclusion criteria: presence of an active ulcerative cutaneous Leishmania lesion, and a positive identification of the parasite from the lesion. (smear microscopy, culture, or PCR); 5–65 years; duration of disease more than 4 weeks; no prior therapy with anti-*Leishmania* drugs; Exclusion: pregnancy; lesion(s) >2,500 mm2; more than 6 cutaneous lesions; mucosal lesion; any acute or chronic illness; concomitant infection; others. CL caused by *L. peruviana*, L. guyanensis, *L. braziliensis*	Imiquimod or placebo vehicle cream three times per week for a total of 9 applications during the 20-day course of treatment with pentavalent antimony. Pentavalent antimony intravenously 20 mg sodium stibogluconate per kg body weight/day for 20 consecutive days to all participants.	Cure rate at 1, 2, 3, 6, 9, 12 months; local side effects.
Neves 2011 (Brazil) [69]	Open-label, controlled, randomized, multicenter at the Tropical Medicine Foundation of Amazonas	Inclusion criteria: weight: greater than 8 kg; clinical findings compatible with CL and positive direct examination (by smear) for *Leishmania*; disease duration: between one and three months of evolution; number of lesions: a maximum of six lesions; presence of at least one ulcerated lesion; lack of mucosal involvement and no history, confirmed or not, of cutaneous leishmanial lesion. Exclusion criteria: Prior treatment with pentavalent antimonials or leishmanicidal drugs in the last six months; evidence of cardiac abnormalities; concomitant tuberculosis, leprosy, cancer, diabetes mellitus or other serious illness; Uncontrolled hypertension; or peripheral vascular involvement; pregnancy; others. CL caused by *L. guyanensis*, and *L braziliensis* was identified.	Pentavalent antimonial at 15 mg/kg/day for 20 days, administered intravenously (IV) or intramuscularly (IM). Pentamidine - three doses of 4 mg/kg were administered every 72 hours via deep intramuscular injection with the patient in a supine position. The maximum dose was 300 mg/dose. Amphotericin B –1 mg/kg/day IV for 20 days. On the first two days, the maximum low dose was (0.5 mg/kg/day). These first two doses were not considered in the calculation of the twenty days of treatment. Rescue treatment: pentamidine isethionate,	Cure rate at 30, 60 and 180 days; rescue treatment; adverse events.
Newlove 2011 (Brazil) [72]	Double-Blind, Placebo-Controlled Trial at the state of Bahia, Brazil	Inclusion criteria: Cutaneous leishmaniasis diagnosed by a typical ulcer and a positive intradermal antigen test; 13–50 years; a maximum of three ulcers; lesion diameter 5–50 mm; and a period of 15 to 60 days from the onset of the ulcer. Exclusion criteria: prior history of CL or Sb v or helminths use; mucosal or disseminated disease; pregnancy; others. CL caused by *L. braziliensis*.	Albendazole (400 mg), ivermectin (200 µg/kg), and praziquantel (50 mg/kg) in an oral formulation at Days 0 and 30 and placebo at Day 60. The control group received placebo. These patients were also treated with the appropriate oral antihelminthic based on parasitological assay results on the 60-day visit. All patients were treated with intravenous pentavalent antimony (Glucantime) at 20 mg/kg/.	Cure rate at 90 days; time to cure; adverse events
Rubiano 2012 (Colombia) [70]	Multicenter, open-label, randomized clinical trial at conducted in 3 geographic locations in Colombia.	Inclusion criteria: children aged 2–12 years with parasitologically confirmed cutaneous leishmaniasis. Exclusion criteria were weight <10 kg, mucocutaneous disease, use of anti-Leishmania medications during the month prior to diagnosis, medical history of cardiac, renal, or hepatic disease, menarche, and others. *L. panamensis* and *L. guyanensis* predominated; few *L. braziliensis.*	Meglumine antimoniate (81 mg Sb/mL) at 20 mg Sb/kg/d intramuscular for 20 consecutive days. Miltefosine (10 mg miltefosine/capsule) at 1.5–2.5 mg/kg/d by mouth during 28 consecutive days, divided into 2 or 3 daily doses.	Cure rate Therapeutic failure during 26 weeks. Parasitologic response; adverse events.
Soto 2008 (Bolivia) [77]	Open-label, randomized clinical trial in Palos Blancos, Bolivia	Inclusion criteria: a skin ulcer confirmed to be caused by leishmania by visualization of parasites in lesion material by Giemsa staining; >12 years of age Exclusion criteria: mucosal disease or anti-leishmanial therapy for at least 6 months; significant concomitant disease; pregnancy or lactation. *L. panamensis, L. guyanensis and L. braziliensis* were identified.	Oral miltefosine 2.5 mg/kg/d for 28 days. Intramuscular pentavalent antimony (glucantime, 20 mg/kg/d) for 20 days.	Cure rate at 1, 3, and 6 months; adverse events
Velez 2010 (Colombia) [75]	Randomized, open-label phase III clinical in five military health establishments located in central, northeast, and southern Colombia	Inclusion criteria: confirmed parasitological diagnosis of leishmaniasis; received no treatment of the current infection during the past 6 weeks; normal renal, hepatic, pancreatic, and hematological functions. Exclusion criteria: serious concomitant illnesses; lesions with mucosal involvement; Disseminated cutaneous leishmaniasis (presence of 10 or more cutaneous lesions and a negative Montenegro skin test). *L. panamensis* and *L. brazililensis* were identified.	Miltefosine 50 mg orally three times per day for 28 days. Meglumine antimoniate intramuscularly at a dose of 20 mg/kg body weight per day for 20 days	Cure rate 6 weeks, 3 months, and 6 months after; failure; recurrence; reinfection Rescue therapy

### Statistical Analysis

We present a summary of main findings from the Cochrane review as well as an update of the evidence provided by new identified trials. We used the RevMan 5.1 software from the Cochrane Collaboration to perform the statistical analysis. For dichotomous primary outcomes the results, expressed as relative risk (RR) and 95% confidence intervals (CI), were calculated using the Mantel–Haenszel random effects model. For the pooled analysis we calculated the I square (I^2^) statistic that describes the percentage of total variation across studies attributed to heterogeneity [30]; low, moderate, and high levels of heterogeneity are roughly estimated as I^2^ values of 25%, 50%, and 75%, respectively. PRISMA checklist is included as supplementary file (Supporting Information S2).

## Results

### Characteristics of Studies

**Figure 1:**
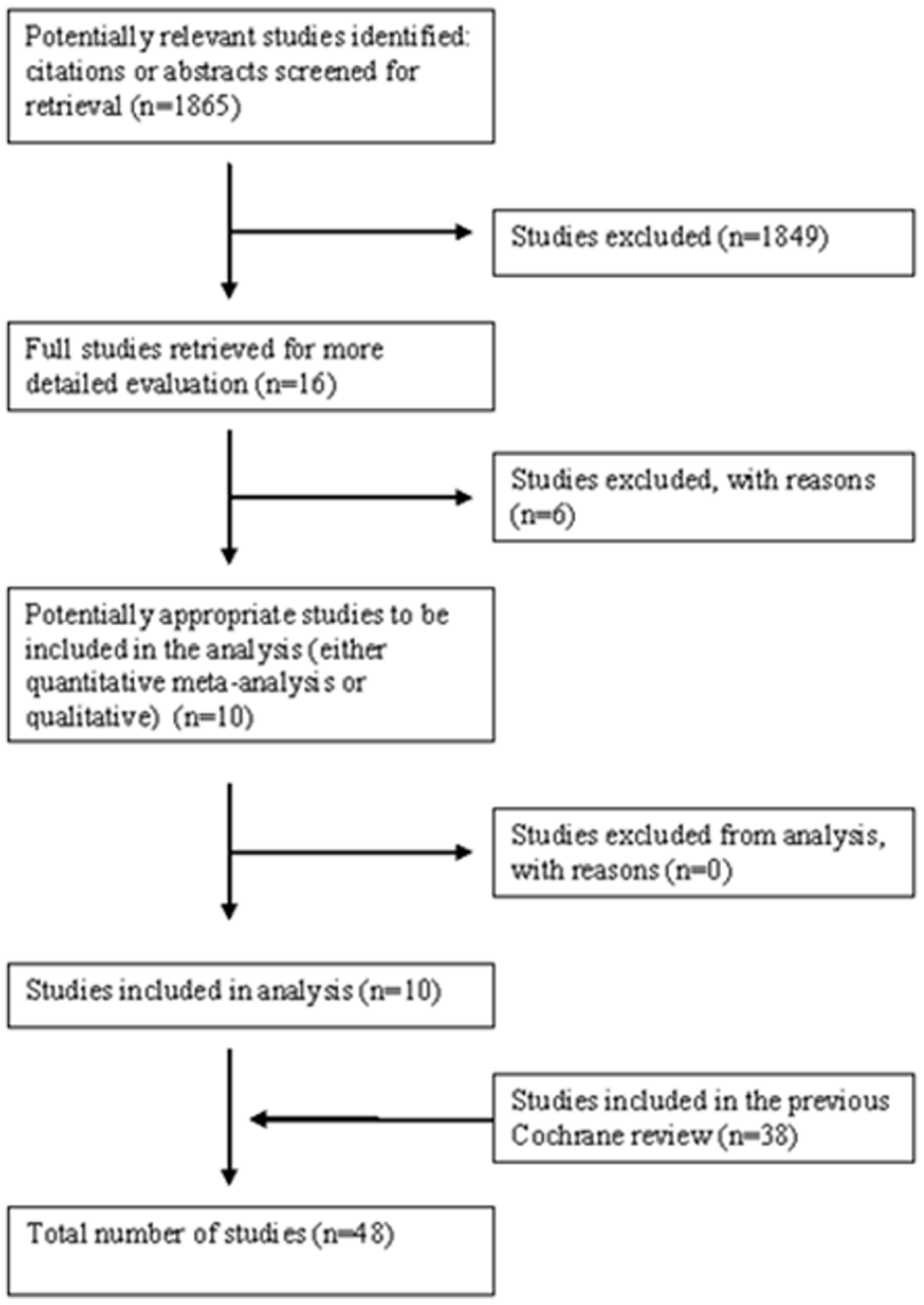
Flow Diagram from a Systematic Review.

The Cochrane review published in 2009 identified 38 randomized controlled [31]–[68] trials. We identified 1865 references of interest (Figure 1) through the literature search and deemed relevant 16 studies on CL or ML [69]–[84]. We included and analyzed 10 new RCTs (Table 1); excluded references are available in Table 2. Four RCTs were conducted in Brazil [69], [72]–[74], four in Colombia [70], [71], [75], [81], one in Bolivia [77], and Peru [76]. The *Leishmania* species responsible for infection were identified in most studies (Table 1) [69]–[77], [81] The follow-up time ranged from 3 months to 1 year. Six references did not comply with eligibility criteria and were excluded [78]–[80], [82]–[84].

**Table 2:** Characteristics of excluded studies.

Motta 2011 [80]	Alternating allocation system
Soto 2009 [78]	Extended follow-up period of another trial; Authors re-treated the patients who initially failed treatment with 6 weeks of therapy, and treated 21 new patients with 6 weeks of therapy.
Sousa 2011 [79]	Not randomized trial
Llanos-Cuentas 2010 [82]	Evaluate the safety and immunogenicity of the LEISH-F1+MPL-SE vaccine when used in combination with sodium stibogluconate for the treatment of mucosal leishmaniasis.
Nascimento E 2010 [83]	Evaluate the safety and immunogenicity of the LEISH-F1+MPL-SE vaccine when used in combination with meglumine antimoniate for the treatment of cutaneous leishmaniasis.
Garcia 2009 [84]	Conference publication of López-Jaramillo 2010 RCT

### Assessment of Risk of Bias

Overall the quality of the reporting and design of the RCTs was moderate to good (Table 3). Nine out of ten RCTs were judged as having low risk of bias for sequence generation; only one was considered having unclear risk of bias [77]. Five RCTs had low risk of bias for allocation concealment [70], [71], [75], [76], [81]. Two studies were placebo controlled trials The majority of trials provided a sample size framework and a scientific rationale for the sample size determination [70]–[76].

**Table 3:** Risk of bias assessment.

**Reference**	**Random sequence generation**	**Allocation** **concealment**	**Blinding (Objective outcomes)**	**Blinding** **(Subjective** **outcomes)**	**Incomplete outcome data and withdrawals (Objective outcomes)**	**Incomplete outcome data and withdrawals (Subjective outcomes)**	**Free of selective reporting?**	**Other sources of bias and commentaries**
Chrusciak-Talhari 2011 [73]	Low	Unclear	Low “Standardized digital photograph”	Unclear (for patient reported adverse events)	Low	NA	Low: retrospective registration: NCT00600548; Authors presented results on all outcome measures that were pre specified as relevant	Low: funding: public (FINEP and CNPq./Brazil)
Lopez 2012 [71]	Low: generated list in blocks of eight using EpiInfo	Low: “Only the clinical coordinator of the study had access to the list and was in charge of allocating treatments”	Unclear: no description of the measurement process	Low: the assessment of side effects was done according to the Common Terminology Criteria for Adverse Events	Low	Low	Low: retrospective registration: NCT00471705; authors presented results on all outcome measures that were pre specified as relevant	Low: funding, Public. Social Protection Ministry of the Republic of Colombia
Lopez-Jaramillo 2010 [81]	Low: randomization list using a computer program.	Low: randomization process was blinded and centralized. The assigned code was reported to the monitoring nurse who had no contact with the participants.	Unclear: no further description of the measurement process nor blinding methods	Unclear: no further description of the measurement process nor blinding methods	Unclear: 35/178 lost to follow up. Difference according to groups.	Unclear: 35/178 lost to follow up. Difference according to groups.	Low: authors presented results on all outcome measures that were pre specified as relevant	Low: funding: Public. Institute for Science and Technology “COLCIENCIAS
Machado 2010 [74]	Low: randomization list using a computer program.	Unclear	Low: “The area involved was calculated as the product of the two measurements. A standardized digital photograph was also taken from each patient’s lesions at the same time points”	Unclear	Low	Low	Low: Retrospective registration: NCT00600548; Authors presented results on all outcome measures that were pre specified as relevant	Low: Funding: Mix Brazilian National Research Council (CNPq). Miltefosine was supplied by Aeterna Zentaris GmbH.
Miranda-Verastegui 2009 [76]	Low: randomization list using a computer program.	Low: “Numbers and corresponding treatment packages were prepared so that both subjects and study investigators were blind to treatment allocation throughout the study”.	Low: both creams were identical in appearance. Standardized photograph of each lesion	Low: both creams were identical in Standardized photograph of each lesion	Low	Low	Low: prospectively registered: NCT00257530; authors presented results on all outcome measures that were pre specified as relevant	Low: Funding: mix. Drugs for Neglected Diseases Initiative (not-for-profit organization). Pharmaceuticals Inc. provided the randomized allocation of imiquimod and placebo creams at no cost
Neves 2011 [69]	Low: list of random distribution generated by a biostatistician	Unclear: not reported	Unclear	Unclear	Unclear: 75.7% of the patients randomized to Amphotericin B refused to continue in the study when they learned that they would need to come to the hospital for at least 20 days	Unclear: 75.7% of the patients randomized to Amphotericin B refused to continue in the study when they learned that they would need to come to the hospital for at least 20 days. 8.1% and 5.4% were loss to follow up in in the meglumine and the pentamidine groups respectively	Unclear: some pre specified outcomes were not considered (i.e. relapse); - The trials was not not registered.	Low: fundings: Financiadora de Estudos e Projetos (Research and Projects Financing) of the Ministry of Science and Technology - FINEP.
Newlove 2011 [72]	Low: randomization table	Unclear: sealed envelopes (opaque or numerated not reported)	Low: placebo was identical in form, color, and number to treatment	Low: placebo was identical in form, color, and number to treatment	Low: no loss to follow up	Low: no loss to follow up	Unclear: prospectively registered: NCT00469495; some pre specified outcomes were not considered (i.e. relapse);	Low: funding: public. NIH/FIC and NIH/NIAID
Rubiano 2012 [70]	Low: computerized balanced block randomization scheme	Low: coordinating center via phone call	Low: to eliminate ascertainment bias, treatment outcome was determined by a masked evaluator using standardized photographs of lesions.	Low: adverse events were identified by study personnel using a structured questionnaire and classified according to Common Terminology Criteria for Adverse Events	Low	Low	Low: prospective registration NCT00487253; authors presented results on all outcome measures that were pre specified as relevant	Low: funding: mix Departamento Administrativo de Ciencia, Tecnologia e Innovacion (COLCIENCIAS); National Institute of Allergy and Infectious Diseases International Collaborations in Infectious Disease Research Program; Fogarty Global Infectious Diseases Research Training Program
Soto 2008 [77]	Unclear: not reported	Unclear: not reported	Unclear	Unclear	Low	Low	Unclear: prospective registration: NCT00233545; not all pre specified outcomes were reported.	Low: funding: NGO AB Foundation
Velez 2010 [75]	Low: list generated randomly in blocks of eight (EpiInfo)	Low: only the study coordinator had access to the list	Unclear	Unclear: adverse events were evaluated according to standard criteria	Low: 14.7% were lost in the miltefosine group while 12.6% were lost in the meglumine antimoniate group. Reasons were explained.	Low	Low: not registered; authors presented results on all outcome measures that were pre specified as relevant	Low: funding: public Ministerio de la Protección Social de la República de Colombia

### Effects of Interventions

#### Miltefosine vs meglumine antimoniate

**Figure 2:**
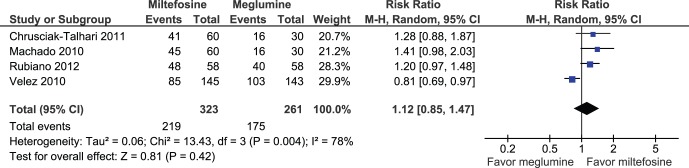
Meta-analysis of four RCTs assessing miltefosine compared to meglumine antimoniate in the complete cure rate at 6 months of follow up.

**Figure 3:**
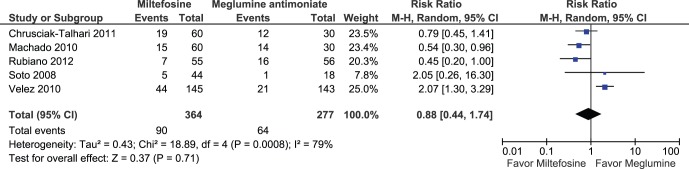
Meta-analysis of five studies assessing miltefosine compared to meglumine antimoniate in clinical failure at 6 months of follow up.

When we pooled four RCTs, miltefosine was not significantly different from meglumine antimoniate in the complete cure rate at 6 months (584 participants; Intent to treat (ITT); RR: 1.12; 95% CI: 0.85 to 1.47; I^2^: 78%; Figure 2) [70], [73]–[75]. Meta-analysis of five studies found no significant difference between miltefosine compared to meglumine antimoniate in clinical failure at 6 months (5 RCT; 641 participants; ITT; RR: 0.88; 95% CI: 0.44 to 1.74; I^2^: 79%; Figure 3) [70], [73]–[75], [77]. Similar findings were found when assessing children in three RCTs (176 participants; RR: 1.16; 95% CI: 0.96 to 1.40; I^2^: 0%) [70], [73], [74], and when evaluating relapses in three RCTs [74], [75], [77].

When considering *Leishmania* species, two studies that mostly included *L. panamensis* and *L. guyanensis* found a significant difference in the rate of complete cure favoring miltefosine at 6 months (2 RCTs, 206 participants; ITT; RR: 1.22 95% CI: 1.02 to 1.46; I^2^: 0%) [70], [73]. One RCT focusing on *L. braziliensis* [74] found a non-significant difference in the rates of complete cure at 6 months favoring miltefosine in Brasil (ITT; RR: 1.41; 95% CI: 0.98 to 2.03) (while another RCT found a significant difference favoring meglumine antimoniate in Colombia (ITT; RR: 0.81; 95% CI: 0.69 to 0.97) [75] meta-analysis of both RCT found no significant difference between group of treatment. Two RCTs assessing failure of treatment at 6 months in *L. guyanensis* found no significant difference between groups (2 RCT; 92 participants; RR: 0.89; 95% CI: 0.32 to 2.48; I^2^: 36%).

In addition, no significant difference was found in serious adverse events rates when combining four studies during follow-up (582 participants; ITT; OR: 1.55; 95% CI: 0.23 to 10.56; I^2^: 0%) [70], [73]–[75].

#### Anthelminthic therapy versus placebo (pentavalent antimony in both arms)

One study [72] found no significant differences in overall time to cure and clinical failure at 3 months between groups. Overall, adverse events (only grade 1 and 2 events were observed) were reported in 60% of patients in both groups.

#### Meglumine antimoniate vs pentamidine

We included one study that evaluated intravenous meglumine antimony compared with intramuscular pentamidine in Brazil [69]. The Cochrane systematic review identified two additional RCTs [32], [40]. Meta-analysis of two RCTs found no significant differences between groups in the rate of complete cure after 6 months of follow-up; however, statistical heterogeneity was very high (I^2^:90%). One RCT [32] found that meglumine antimoniate was superior to pentamidine in the rate of complete cure in the treatment of *L. braziliensis* (80 particpants, ITT RR 2.21 95% CI: 1.41–3.49), while another RCT [69] assessing *L. guyanensis* did not find any significant difference. Another RCT [40] also did not found any significant difference in the rate of failure between treatment groups after one year of follow-up *(L. braziliensis)*. No significant differences between groups were found when assessing serious adverse events.

#### Imiquimod versus placebo (pentavalent antimony in both arms)

**Figure 4:**
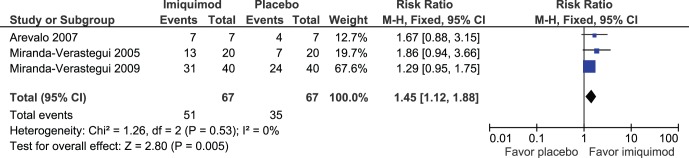
Meta-analysis of the three studies evaluating imiquimod compared to placebo in the rate of complete cure at 3 months of follow up.

We included one RCT assessing the effects of imiquimod compared to placebo in Peru [76]. Two additional RCTs were incorporated from the Cochrane systematic review [35], [52].Although meta-analysis of the three studies found a significant difference favoring the treatment group in the rate of complete cure at 3 months of follow up (134 participants; ITT; RR: 1.45; 95% CI: 1.12 to 1.88; I^2^: 0%; Figure 4) no significant differences were found when combining two RCTs [52], [76] at 6(120 participants; ITT RR 1.22 95% CI 0.94 to 1.59; I2 0%), and 12 months (120 participants; ITT; RR: 1.09; 95% CI: 0.73 to 1.62; I^2^: 58%). Additionally, no significant difference was found when evaluating serious adverse events in two RCTs [52], [76].

#### Nitric oxide releasing patch vs meglumine antimoniate

One study [81] found a significant difference favoring meglumine antimoniate in complete cure (143 participants; RR: 0.40; 95% CI: 0.29 to 0.55). No significant difference was found for serious adverse events. Patients treated with nitric oxide releasing patch presented a significant lower proportion of non-serious adverse events such as fever, headache, myalgia, and arthralgia while those treated with meglumine antimoniate had a significant lower proportion of local rash and pain.

#### Thermotherapy versus meglumine antimoniate

A significant difference favoring meglumine antimoniate was found in the rate of complete cure at 6 months (189 participants; RR: 0.80; 95% CI: 0.68 to 0.95) [71]. However, no significant difference was found when analyzing *L. panamensis* (37 participants; RR: 0.81; 95% CI: 0.54 to 1.21) and *L. braziliensis* (65 participants; RR: 0.80; 95% CI: 0.59 to 1.10) [71]. An RCT [53] from the Cochrane review found no significant difference between groups.

A summary of the main findings from the previous Cochrane review and the updated studies can be found in Table 4.

**Table 4:** Main finding from Cochrane review and this update for complete cure.

Intervention	Comparator	Effect measure (RR, OR, mean) [95%confidence interval] [Heterogeneity]	Follow up period	Parasite species	References
Miltefosine	Meglumineantimoniate	ITT RR 1.12 (0.85–1.47); N = 584; I2 78%	6 months	*L. braziliensis,* *L. panamensis and* *L. guyanensis*	Chrusciak,2011 [73];Machado,2010 [74];Rubiano,2012 [70];Velez,2010 [75]
Miltefosine*	Meglumineantimoniate	ITT RR 1.22 (1.02–1.46); N = 206; I2 0%	6 months	*L. panamensis and* *L. guyanensis*	Chrusciak,2011 [73];Rubiano,2012 [70]
Miltefosine	Meglumineantimoniate*	ITT; RR 0.81 (0.69–0.97)	6 months	*L. braziliensis,* *L panamensis*	Velez,2010 [75]
Imiquimod*	Placebo	ITT RR 1.45 (1.12–1.88); N = 134; I2 0%;	3 months	*L. peruviana,* *L. guyanensis,* *L. braziliensis*	Miranda V 2005 [52];MirandaV 2009 [76]Arevalo 2007 [35]
Imiquimod	Placebo	ITT RR 1.09 (0.73–1.62); N = 120; I^2^ 58%	1 year	*L. peruviana,* *L. guyanensis,* *L. braziliensis*	Miranda V 2005 [52];MirandaV 2009 [76]
Pentamidineisethionate	Meglumineantimoniate	ITT Yes; 6 of 80 (7.5%); N = 70	6 months	*Leishmania* *braziliensis*	Andersen,2005 [32]
Meglumineantimoniate	Meglumineantimoniate	ITT = No; 4 of 43 (9.3%); N = 43	2 years	No information	Figueiredo,1991 [42]
Sodiumstibogluconate	Sodiumstibogluconate	ITT = No; 5 of 40 (12.5%); N = 40	1 year	*L. braziliensis*	Franke,1994 [43]
Thermotherapy	Meglumineantimoniate	ITT = No; 1 of 37 (2.7%); N = 37	Treatmentend	*L. braziliensis*	Lobo,2006 [47]
Oralpentoxyfilline+Sodiumstibogluconate	Placebo+Sodiumstibogluconate	ITTI = Yes uninformed; N = 23	4 months	*L. braziliensis*	Machado,2007 [48]
Meglumineantimoniate	Thermotherapy	ITT = Yes, uninformed; N = 66	2 months	*L. braziliensis* and*L. mexicana*	Navin1990 [53]
Oral Ketoconazole	Sodiumstibogkuconate	ITT = No; 7 of 120 (5.83%); N = 120	2 months	*L. braziliensis* and*L. mexicana*	Navin1992 [54]
Meglumineantimoniate	Meglumineantimoniate	ITT = Yes; uninformed; N = 23	2 months	*L. braziliensis*	Oliveira-Neto,1997 [56]
Sodiumstibogluconate	Sodiunstibogluconate	ITT = Yes, uninformed;	Treatmentend	*L. braziliensis (27)* *L. mexicana (9)* *L. chagasi (3)*	Oster,1995 [57]

* Significant difference favoring this intervention.

## Discussion

The present update on the treatment of American CL showed an increase in the number of papers published during the past 3 years and an improvement of the quality of the studies. The 10 RCTs included in this systematic review represent more than 25% of the 38 studies included in the Cochrane Review 2009, which covered a period of 25 years. The scope of this systematic review took into consideration the main challenges faced in this specific therapeutic field, mainly, the parasite diversity observed across the continent, the variety of therapeutic interventions currently in use and the importance of the quality of the included reports which were submitted to a stringent evaluation of risk of bias.

Miltefosine, considered as the first effective oral treatment for cutaneous leishmaniasis has been used at a dose of 2.5 mg/kg weight, with cure rates which vary both according to species and to the geographic location where the studies have been performed [85]. Adverse effects include vomiting, nausea, kinetosis and headache, and elevation of creatinine and aminotransferase levels [86]. Meglumine antimoniate has been widely used for the treatment of ACL. The currently recommended dose is 20 mg/kg of body weight/day for 20 days [5]. For these reasons, meglumine antimoniate is frequently used as the comparator in clinical trials of new treatments for ACL [86].

The attempt to summarize the effect of miltefosine compared to meglumine antimoniate included four studies and indicated no difference between treatments [70], [73]–[75]. However, the heterogeneity measure was high and a careful observation of data showed that one study, the one with the largest sample size [75], demonstrated an inconsistent effect compared to the other three [70], [74], [87]. This inconsistent study concluded with statistical significance for the inferiority of miltefosine in patients infected with *L. braziliensis* or *L. panamensis* and the other three concluded for the lack of difference between both drugs. Furthermore, the two studies conducted in Brazil included patients with just one parasite species in each study, *L. braziliensis* [74] and *L. guyanensis* [73]. However, the apparent consistency between the Brazilian studies needs to be taken with caution because of the differences of therapeutic response rate observed between *L. braziliensis* and *L. guyanensis* infected patients as previously reported in a quasiexperimental study [88].The Colombian studies are more difficult to analyze because of the inclusion of a mixture of patients infected with at least two parasite species in each study [70], [75]. Just one species, *L. panamensis*, was common to both studies. The therapeutic response variation observed in patients from different geographical areas could be at least partially explained by the diversity of parasite species that was well documented in those four trials but further evidences are needed to conclude that parasite species was determinant of the therapeutic response and homogeneous across different geographical regions [5]. Recently, data from Peru stimulated the debate on the role of parasite species on therapeutic response to antimonials and raised other hypotheses to explain the differences observed between Peruvian and Brazilian cases infected *with L. braziliensis* or *L. guyanensis* [89].

Overall, caution needs to be applied to the summary estimates related to the comparison of miltefosine with meglumine antimoniate and these data deserve proper contextualization for each of the specific scenarios where the evidences were produced.

Anthelminthic therapy versus placebo, both associated with standard treatment with antimonials, was evaluated in just one RCT showing no significant difference [72]. The rational for this approach is based on the possible influence of helminths parasites on the modulation of the immune response against leishmaniasis. This study deserves attention and further investigation because of the small sample size and the unexpected worse response observed (although not statistically significant) in the group submitted to anthelminthic therapy.

Pentamidine isethionate has been used for the treatment of cutaneous leishmaniasis at a dose of 2–4 mg/kg/day with 2 to 4 applications on alternate days. Frequent adverse effects include musculoskeletal pain, anorexia, abdominal pain, nausea, vomiting, headache, asthenia and fatigue. Pentamidine can also cause hypoglycemia, which can sometimes be severe, and insulin-dependent diabetes mellitus [86], [90].

The attempt to meta-analyze the evidences that compared pentamidine against antimonials was troublesome because of the high heterogeneity observed which again could be due to the diversity of parasite species causing the diseases and the geographical variation in therapeutic response already mentioned above.

Imiquimod stimulates the production of nitric oxide by macropahges, which decreases the number of parasites in vitro [85]. Topical imiquimod has been used in combination with pentavalent antimonials for treatment of cutaneous leishmaniasis [35], [52]. In spite of the lack of significant differences observed for the six-month and 12-month outcomes the result of the summary estimate indicating the benefit of imiquimod improving the initial response obtained at the three months with antimonials deserves more attention. Sometimes the time to obtain clinical cure is not included at least as a secondary endpoint to evaluate the usefulness of therapeutic interventions. However reducing time-to-cure period could be interesting to save costs for the health system.

The included evidence on nitric oxide is not encouraging to put more resources in such type of intervention and could be considered as a proof of principle of lack of therapeutic effect.

The only new evidence on thermotherapy demonstrated the inferiority of this intervention in patients infected with *L. panamensis* or *L. braziliensis*. Subgroup analysis was strongly affected by the small sample size, but both subgroups maintained a consistent direction of the effect with point estimates favoring antimonial treatment.

The rationale for administering local treatments, which include thermotherapy, intralesional administration of pentavalent antimonials, and topical treatments, is that the risk of developing a mucosal form is low, not necessarily prevented by systemic treatment, and localized treatments are better tolerated and have less frequent and severe adverse effects as compared to systemic treatments [91]. However, there is a need to standardize and evaluate the efficacy of localized and topical treatments for cutaneous leishmaniasis and to develop recommendations for their use.

This study also confirmed the lack of RCTs in patients with the mucosal form of the disease. This is a relevant “negative” result which is widely recognized as a pitfall in the health care offered to patients with leishmaniasis. This is a neglected aspect that remains waiting for the organization of a multicentric initiative to develop RCTs to prove the efficacy of the current therapeutic options.

The main limitation of the present systematic review is the lack of a larger number of studies to perform the meta-analysis taking into consideration the already known characteristics which affect the prognosis of CL. Small sample size is still a problem in RCTs for leishmaniasis. Meta-analysis could help if the scenarios were homogeneous but this is not the case as already demonstrated for different parasite species and the geographical variation in response to treatment. In addition, some RCT s had short follow-up periods in which relapses could easily be missed in a chronic condition like MCL/CL. Future studies should consider longer follow up period [92].

Data on parasite species appears to be more commonly registered in recent trials but some patients are still included and treated without this information. The need for species-specific parasitological diagnosis of patients enrolled in clinical trials needs to be stressed. Recent development of molecular tools allows species identification with some effort but fortunately, nowadays, the success in parasite isolation and culture, which is more troublesome, is no longer required [93].

Although there are no simple and cheap assays to evaluate parasite resistance in vitro and that such type of evaluation requires parasite isolation, it would be reasonable to nest into RCTs a subgroup analysis of resistance to the specific drugs which are under evaluation, mainly in those scenarios where lack of therapeutic response is high [94], [95].

Finally, the lack of registry of other potential prognostic factors such as immunological status [96], co-morbidity, [72] age-related pharmacokinetics) [97] which could explain the observed differences between studies and regions deserves more attention and a minimum set of variables with prognosis potential needs to be discussed for further trials in order to enrich the comprehension of the observed variability. This could be as important as the use of standardized outcomes and time to main and secondary outcomes.

In conclusion the present updated systematic review revealed that a lot of work needs to be done to achieve a strong evidence to recommend specific treatments against cutaneous leishmaniasis. There is still a need for well conducted RCT to assess the effectiveness and safety of different anti-Leishmania alternatives drugs. As compared with the Cochrane review [29], studies included in this update had lower risk of bias and reported information in a more standardize manner. Local or regional evidences should be obtained taking into consideration parasite species diversity and other prognostic factors to make valuable evidence-based recommendations. Mucosal disease is the more neglected form of CL and a multicentric trial should be urgently considered.

## Supporting Information

Supporing Information S1 **Search strategies.**
(DOC)

Supporting Information S2 **PRISMA 2009 Checklist – Leishmaniasis.**
(DOC)

## References

[1] Ashford RW, Desjeux P, Deraadt P (1992) Estimation of population at risk of infection and number of cases of Leishmaniasis. Parasitol Today 8: 104–105. 15463585 10.1016/0169-4758(92)90249-2

[2] Copeland HW, Arana BA, Navin TR (1990) Comparison of active and passive case detection of cutaneous leishmaniasis in Guatemala. Am J Trop Med Hyg 43: 257–259. 2221220 10.4269/ajtmh.1990.43.257

[3] Yadon ZE, Quigley MA, Davies CR, Rodrigues LC, Segura EL (2001) Assessment of Leishmaniasis notification system in Santiago del Estero, Argentina, 1990–1993. Am J Trop Med Hyg 65: 27–30. 11504403 10.4269/ajtmh.2001.65.27

[4] Maia-Elkhoury AN, Carmo EH, Sousa-Gomes ML, Mota E (2007) [Analysis of visceral leishmaniasis reports by the capture-recapture method]. Rev Saude Publica 41: 931–937. 18066464 10.1590/s0034-89102007000600007

[5] World Health Organization (2010) Control of Leishmaniasis: report of the meeting of the WHO Expert commitee on the control of leishmaniases. Geneva: World Health Organization. 949 949.

[6] Alvar J, Velez ID, Bern C, Herrero M, Desjeux P, et al (2012) Leishmaniasis worldwide and global estimates of its incidence. PLoS One 7: e35671. 22693548 10.1371/journal.pone.0035671 PMC3365071

[7] Desjeux P (1992) Human leishmaniases: epidemiology and public health aspects. World Health Stat Q 45: 267–275. 1462660

[8] World Health Organization (1990) Control of the leishmaniases. Report of a WHO Expert Committee. World Health Organization. 1–158 p. 2124015

[9] Turetz ML, Machado PR, Ko AI, Alves F, Bittencourt A, et al (2002) Disseminated leishmaniasis: a new and emerging form of leishmaniasis observed in northeastern Brazil. J Infect Dis 186: 1829–1834. 12447770 10.1086/345772

[10] Grimaldi G Jr, Tesh RB (1993) Leishmaniases of the New World: current concepts and implications for future research. Clin Microbiol Rev 6: 230–250. 8358705 10.1128/cmr.6.3.230 PMC358284

[11] Yadon ZE, Rodrigues LC, Davies CR, Quigley MA (2003) Indoor and peridomestic transmission of American cutaneous leishmaniasis in northwestern Argentina: a retrospective case-control study. Am J Trop Med Hyg 68: 519–526. 12812336 10.4269/ajtmh.2003.68.519

[12] Cerf BJ, Jones TC, Badaro R, Sampaio D, Teixeira R, et al (1987) Malnutrition as a risk factor for severe visceral leishmaniasis. J Infect Dis 156: 1030–1033. 3680989 10.1093/infdis/156.6.1030

[13] Alvar J, Yactayo S, Bern C (2006) Leishmaniasis and poverty. Trends Parasitol 22: 552–557. 17023215 10.1016/j.pt.2006.09.004

[14] Ocampo CB, Ferro MC, Cadena H, Gongora R, Perez M, et al.. (2012) Environmental factors associated with American cutaneous leishmaniasis in a new Andean focus in Colombia. Trop Med Int Health. 10.1111/j.1365-3156.2012.03065.x PMC5079278 22882595

[15] Cardenas R, Sandoval CM, Rodriguez-Morales AJ, Vivas P (2008) Zoonoses and climate variability. Ann N Y Acad Sci 1149: 326–330. 19120241 10.1196/annals.1428.094

[16] Reithinger R, Dujardin JC, Louzir H, Pirmez C, Alexander B, et al (2007) Cutaneous leishmaniasis. Lancet Infect Dis 7: 581–596. 17714672 10.1016/S1473-3099(07)70209-8

[17] Velez I, Agudelo S, Robledo S, Jaramillo L, Segura I, et al (1994) Diffuse cutaneous leishmaniasis with mucosal involvement in Colombia, caused by an enzymatic variant of Leishmania panamensis. Trans R Soc Trop Med Hyg 88: 199. 8036672 10.1016/0035-9203(94)90294-1

[18] Silveira FT, Lainson R, Corbett CE (2004) Clinical and immunopathological spectrum of American cutaneous leishmaniasis with special reference to the disease in Amazonian Brazil: a review. Mem Inst Oswaldo Cruz 99: 239–251. 15273794 10.1590/s0074-02762004000300001

[19] Saldanha ACR, Gama M, M-Elkhoury ANM, Barral A, Bezerril ACR, et al (2009) Clinical Cure In Diffuse Cutaneous Leishmaniasis (DCL) In Brazil. Bahia Gaz méd 79 (Supl.3): 52–61.

[20] Belli A, Garcia D, Palacios X, Rodriguez B, Valle S, et al (1999) Widespread atypical cutaneous Leishmaniasis caused by Leishmania (L.) Chagasi in Nicaragua. Am J Trop Med Hyg 61: 380–385. 10497975 10.4269/ajtmh.1999.61.380

[21] De Lima H, Rodriguez N, Feliciangeli MD, Barrios MA, Sosa A, et al (2009) Cutaneous leishmaniasis due to Leishmania chagasi/Le. infantum in an endemic area of Guarico State, Venezuela. Trans R Soc Trop Med Hyg 103: 721–726. 19150102 10.1016/j.trstmh.2008.11.019

[22] Guerra JA, Prestes SR, Silveira H, Coelho LI, Gama P, et al (2011) Mucosal Leishmaniasis caused by Leishmania (Viannia) braziliensis and Leishmania (Viannia) guyanensis in the Brazilian Amazon. PLoS Negl Trop Dis 5: e980. 21408116 10.1371/journal.pntd.0000980 PMC3050903

[23] Amato VS, Tuon FF, Bacha HA, Neto VA, Nicodemo AC (2008) Mucosal leishmaniasis. Current scenario and prospects for treatment. Acta Trop 105: 1–9. 17884002 10.1016/j.actatropica.2007.08.003

[24] Davies CR, Reithinger R, Campbell-Lendrum D, Feliciangeli D, Borges R, et al (2000) The epidemiology and control of leishmaniasis in Andean countries. Cad Saude Publica 16: 925–950. 11175518 10.1590/s0102-311x2000000400013

[25] Lessa MM, Lessa HA, Castro TW, Oliveira A, Scherifer A, et al (2007) Mucosal leishmaniasis: epidemiological and clinical aspects. Braz J Otorhinolaryngol 73: 843–847. 18278231 10.1016/S1808-8694(15)31181-2 PMC9450602

[26] Tedesqui VL, Calleja GN, Parra R, Pabon JP, Boia MN, et al (2012) Active surveillance of American tegumentary leishmaniasis in endemic areas in rural Bolivia. Rev Soc Bras Med Trop 45: 30–34. 22370825 10.1590/s0037-86822012000100007

[27] Hashiguchi Y, Gomez Landires EA (1991) A review of leishmaniasis in Ecuador. Bull Pan Am Health Organ 25: 64–76. 2054554

[28] Boggild AK, Valencia BM, Veland N, Pilar Ramos A, Calderon F, et al (2011) Non-invasive cytology brush PCR diagnostic testing in mucosal leishmaniasis: superior performance to conventional biopsy with histopathology. PLoS One 6: e26395. 22046280 10.1371/journal.pone.0026395 PMC3203107

[29] Gonzalez U, Pinart M, Rengifo-Pardo M, Macaya A, Alvar J, et al.. (2009) Interventions for American cutaneous and mucocutaneous leishmaniasis. Cochrane Database Syst Rev: CD004834. 10.1002/14651858.CD004834.pub2 19370612

[30] Higgins JPT, Green S (2011) Cochrane Handbook for Systematic Reviews of Interventions Version 5.1.0 [updated March 2011]. The Cochrane Collaboration.

[31] Almeida R, D’Oliveira A Jr, Machado P, Bacellar O, Ko AI, et al (1999) Randomized, double-blind study of stibogluconate plus human granulocyte macrophage colony-stimulating factor versus stibogluconate alone in the treatment of cutaneous Leishmaniasis. J Infect Dis 180: 1735–1737. 10515844 10.1086/315082

[32] Andersen EM, Cruz-Saldarriaga M, Llanos-Cuentas A, Luz-Cjuno M, Echevarria J, et al (2005) Comparison of meglumine antimoniate and pentamidine for peruvian cutaneous leishmaniasis. Am J Trop Med Hyg 72: 133–137. 15741547

[33] Arana BA, Navin TR, Arana FE, Berman JD, Rosenkaimer F (1994) Efficacy of a short course (10 days) of high-dose meglumine antimonate with or without interferon-gamma in treating cutaneous leishmaniasis in Guatemala. Clin Infect Dis 18: 381–384. 8011819 10.1093/clinids/18.3.381

[34] Arana BA, Mendoza CE, Rizzo NR, Kroeger A (2001) Randomized, controlled, double-blind trial of topical treatment of cutaneous leishmaniasis with paromomycin plus methylbenzethonium chloride ointment in Guatemala. Am J Trop Med Hyg 65: 466–470. 11716099 10.4269/ajtmh.2001.65.466

[35] Arevalo I, Tulliano G, Quispe A, Spaeth G, Matlashewski G, et al (2007) Role of imiquimod and parenteral meglumine antimoniate in the initial treatment of cutaneous leishmaniasis. Clin Infect Dis 44: 1549–1554. 17516397 10.1086/518172

[36] Armijos RX, Weigel MM, Calvopina M, Mancheno M, Rodriguez R (2004) Comparison of the effectiveness of two topical paromomycin treatments versus meglumine antimoniate for New World cutaneous leishmaniasis. Acta Trop 91: 153–160. 15234664 10.1016/j.actatropica.2004.03.009

[37] Ballou WR, McClain JB, Gordon DM, Shanks GD, Andujar J, et al (1987) Safety and efficacy of high-dose sodium stibogluconate therapy of American cutaneous leishmaniasis. Lancet 2: 13–16. 2885505 10.1016/s0140-6736(87)93053-4

[38] Convit J, Castellanos PL, Rondon A, Pinardi ME, Ulrich M, et al (1987) Immunotherapy versus chemotherapy in localised cutaneous leishmaniasis. Lancet 1: 401–405. 2880213 10.1016/s0140-6736(87)90116-4

[39] Convit J, Castellanos PL, Ulrich M, Castes M, Rondon A, et al (1989) Immunotherapy of localized, intermediate, and diffuse forms of American cutaneous leishmaniasis. J Infect Dis 160: 104–115. 2659679 10.1093/infdis/160.1.104

[40] Correia D, Macedo VO, Carvalho EM, Barral A, Magalhaes AV, et al (1996) Comparative study of meglumine antimoniate, pentamidine isethionate and aminosidine sulfate in the treatment of primary skin lesions caused by Leishmania (Viannia) braziliensis. Rev Soc Bras Med Trop 29: 447–453. 8966308 10.1590/s0037-86821996000500007

[41] D’Oliveira Junior A, Machado PR, Carvalho EM (1997) Evaluating the efficacy of allopurinol for the treatment of cutaneous leishmaniasis. Int J Dermatol 36: 938–940. 9466205 10.1046/j.1365-4362.1997.00308.x

[42] Figueiredo Kopke LF, Siviero do Vale EC, Grossi Araujo M, Araújo Magalhâes P, Furtado T (1991) Treatment of American Tegumentary Leishmaniasis with N-methylglucamine: double-blind study with doses of 14 mg/kg/day and 28 mg/kg/day of antimoniate [Tratamento da leishmaniose tegumentar americana pelo antimoniato de N-metil-glucamina: Estudo duplo-cego com doses de 14 mg/kg/dia e 28 mg/kg/dia de antimônio]. Anais Brasileiros de Dermatologia 66: 87–94.

[43] Franke ED, Llanos-Cuentas A, Echevarria J, Cruz ME, Campos P, et al (1994) Efficacy of 28-day and 40-day regimens of sodium stibogluconate (Pentostam) in the treatment of mucosal leishmaniasis. Am J Trop Med Hyg 51: 77–82. 8059918 10.4269/ajtmh.1994.51.77

[44] Guderian RH, Chico ME, Rogers MD, Pattishall KM, Grogl M, et al (1991) Placebo controlled treatment of Ecuadorian cutaneous leishmaniasis. Am J Trop Med Hyg 45: 92–97. 1651060 10.4269/ajtmh.1991.45.92

[45] Hepburn NC, Tidman MJ, Hunter JA (1994) Aminosidine (paromomycin) versus sodium stibogluconate for the treatment of American cutaneous leishmaniasis. Trans R Soc Trop Med Hyg 88: 700–703. 7886779 10.1016/0035-9203(94)90237-2

[46] Llanos-Cuentas A, Echevarria J, Cruz M, La Rosa A, Campos P, et al (1997) Efficacy of sodium stibogluconate alone and in combination with allopurinol for treatment of mucocutaneous leishmaniasis. Clin Infect Dis 25: 677–684. 9314461 10.1086/513776

[47] Lobo IM, Soares MB, Correia TM, de Freitas LA, Oliveira MI, et al (2006) Heat therapy for cutaneous leishmaniasis elicits a systemic cytokine response similar to that of antimonial (Glucantime) therapy. Trans R Soc Trop Med Hyg 100: 642–649. 16274713 10.1016/j.trstmh.2005.08.011

[48] Machado PR, Lessa H, Lessa M, Guimaraes LH, Bang H, et al (2007) Oral pentoxifylline combined with pentavalent antimony: a randomized trial for mucosal leishmaniasis. Clin Infect Dis 44: 788–793. 17304449 10.1086/511643

[49] Machado-Pinto J, Pinto J, da Costa CA, Genaro O, Marques MJ, et al (2002) Immunochemotherapy for cutaneous leishmaniasis: a controlled trial using killed Leishmania (Leishmania) amazonensis vaccine plus antimonial. Int J Dermatol 41: 73–78. 11982640 10.1046/j.1365-4362.2002.01336.x

[50] Martinez S, Marr JJ (1992) Allopurinol in the treatment of American cutaneous leishmaniasis. N Engl J Med 326: 741–744. 1738379 10.1056/NEJM199203123261105

[51] Martinez S, Gonzalez M, Vernaza ME (1997) Treatment of cutaneous leishmaniasis with allopurinol and stibogluconate. Clin Infect Dis 24: 165–169. 9114142 10.1093/clinids/24.2.165

[52] Miranda-Verastegui C, Llanos-Cuentas A, Arevalo I, Ward BJ, Matlashewski G (2005) Randomized, double-blind clinical trial of topical imiquimod 5% with parenteral meglumine antimoniate in the treatment of cutaneous leishmaniasis in Peru. Clin Infect Dis 40: 1395–1403. 15844060 10.1086/429238

[53] Navin TR, Arana BA, Arana FE, de Merida AM, Castillo AL, et al (1990) Placebo-controlled clinical trial of meglumine antimonate (glucantime) vs. localized controlled heat in the treatment of cutaneous leishmaniasis in Guatemala. Am J Trop Med Hyg 42: 43–50. 2405727 10.4269/ajtmh.1990.42.43

[54] Navin TR, Arana BA, Arana FE, Berman JD, Chajon JF (1992) Placebo-controlled clinical trial of sodium stibogluconate (Pentostam) versus ketoconazole for treating cutaneous leishmaniasis in Guatemala. J Infect Dis 165: 528–534. 1311351 10.1093/infdis/165.3.528

[55] Neva FA, Ponce C, Ponce E, Kreutzer R, Modabber F, et al (1997) Non-ulcerative cutaneous leishmaniasis in Honduras fails to respond to topical paromomycin. Trans R Soc Trop Med Hyg 91: 473–475. 9373659 10.1016/s0035-9203(97)90290-x

[56] Oliveira-Neto MP, Schubach A, Mattos M, Goncalves-Costa SC, Pirmez C (1997) Treatment of American cutaneous leishmaniasis: a comparison between low dosage (5 mg/kg/day) and high dosage (20 mg/kg/day) antimony regimens. Pathol Biol (Paris) 45: 496–499. 9309267

[57] Oster CN, Chulay JD, Hendricks LD, Pamplin CL 3rd, Ballou WR, et al (1985) American cutaneous leishmaniasis: a comparison of three sodium stibogluconate treatment schedules. Am J Trop Med Hyg 34: 856–860. 2994500 10.4269/ajtmh.1985.34.856

[58] Palacios R, Osorio LE, Grajalew LF, Ochoa MT (2001) Treatment failure in children in a randomized clinical trial with 10 and 20 days of meglumine antimonate for cutaneous leishmaniasis due to Leishmania viannia species. Am J Trop Med Hyg 64: 187–193. 11442216 10.4269/ajtmh.2001.64.187

[59] Saenz RE, Paz HM, Johnson CM, Narvaez E, de Vasquez AM (1987) Evaluation of the effectiveness and toxicity of pentostam and glucantime in the treatment of cutaneous leishmaniasis. Rev Med Panama 12: 148–157. 2827242

[60] Saenz RE, Paz H, Berman JD (1990) Efficacy of ketoconazole against Leishmania braziliensis panamensis cutaneous leishmaniasis. Am J Med 89: 147–155. 2166429 10.1016/0002-9343(90)90292-l

[61] Santos JB, de Jesus AR, Machado PR, Magalhaes A, Salgado K, et al (2004) Antimony plus recombinant human granulocyte-macrophage colony-stimulating factor applied topically in low doses enhances healing of cutaneous Leishmaniasis ulcers: a randomized, double-blind, placebo-controlled study. J Infect Dis 190: 1793–1796. 15499535 10.1086/424848

[62] Soto J, Grogl M, Berman J, Olliaro P (1994) Limited efficacy of injectable aminosidine as single-agent therapy for Colombian cutaneous leishmaniasis. Trans R Soc Trop Med Hyg 88: 695–698. 7886777 10.1016/0035-9203(94)90235-6

[63] Soto J, Fuya P, Herrera R, Berman J (1998) Topical paromomycin/methylbenzethonium chloride plus parenteral meglumine antimonate as treatment for American cutaneous leishmaniasis: controlled study. Clin Infect Dis 26: 56–58. 9455509 10.1086/516267

[64] Soto JM, Toledo JT, Gutierrez P, Arboleda M, Nicholls RS, et al (2002) Treatment of cutaneous leishmaniasis with a topical antileishmanial drug (WR279396): phase 2 pilot study. Am J Trop Med Hyg 66: 147–151. 12135285 10.4269/ajtmh.2002.66.147

[65] Soto J, Valda-Rodriquez L, Toledo J, Vera-Navarro L, Luz M, et al (2004) Comparison of generic to branded pentavalent antimony for treatment of new world cutaneous leishmaniasis. Am J Trop Med Hyg 71: 577–581. 15569787

[66] Soto J, Arana BA, Toledo J, Rizzo N, Vega JC, et al (2004) Miltefosine for new world cutaneous leishmaniasis. Clin Infect Dis 38: 1266–1272. 15127339 10.1086/383321

[67] Velez I, Agudelo S, Hendrickx E, Puerta J, Grogl M, et al (1997) Inefficacy of allopurinol as monotherapy for Colombian cutaneous leishmaniasis. A randomized, controlled trial. Ann Intern Med 126: 232–236. 9027276 10.7326/0003-4819-126-3-199702010-00010

[68] Llanos-Cuentas A, Echevarria J, Seas C, Chang E, Cruz M, et al (2007) Parenteral aminosidine is not effective for Peruvian mucocutaneous leishmaniasis. Am J Trop Med Hyg 76: 1128–1131. 17556623

[69] Neves LO, Talhari AC, Gadelha EP, Silva Junior RM, Guerra JA, et al (2011) A randomized clinical trial comparing meglumine antimoniate, pentamidine and amphotericin B for the treatment of cutaneous leishmaniasis by Leishmania guyanensis. An Bras Dermatol 86: 1092–1101. 22281895 10.1590/s0365-05962011000600005

[70] Rubiano LC, Miranda MC, Muvdi Arenas S, Montero LM, Rodriguez-Barraquer I, et al (2012) Noninferiority of miltefosine versus meglumine antimoniate for cutaneous leishmaniasis in children. J Infect Dis 205: 684–692. 22238470 10.1093/infdis/jir816 PMC3266136

[71] Lopez L, Robayo M, Vargas M, Velez I (2012) Thermotherapy. An alternative for the treatment of American cutaneous leishmaniasis. Trials 13: 58. 22594858 10.1186/1745-6215-13-58 PMC3441257

[72] Newlove T, Guimaraes LH, Morgan DJ, Alcantara L, Glesby MJ, et al (2011) Antihelminthic therapy and antimony in cutaneous leishmaniasis: a randomized, double-blind, placebo-controlled trial in patients co-infected with helminths and Leishmania braziliensis. Am J Trop Med Hyg 84: 551–555. 21460008 10.4269/ajtmh.2011.10-0423 PMC3062447

[73] Chrusciak-Talhari A, Dietze R, Chrusciak Talhari C, da Silva RM, Gadelha Yamashita EP, et al (2011) Randomized controlled clinical trial to access efficacy and safety of miltefosine in the treatment of cutaneous leishmaniasis Caused by Leishmania (Viannia) guyanensis in Manaus, Brazil. Am J Trop Med Hyg 84: 255–260. 21292895 10.4269/ajtmh.2011.10-0155 PMC3029178

[74] Machado PR, Ampuero J, Guimaraes LH, Villasboas L, Rocha AT, et al (2010) Miltefosine in the treatment of cutaneous leishmaniasis caused by Leishmania braziliensis in Brazil: a randomized and controlled trial. PLoS Negl Trop Dis 4: e912. 21200420 10.1371/journal.pntd.0000912 PMC3006132

[75] Velez I, Lopez L, Sanchez X, Mestra L, Rojas C, et al (2010) Efficacy of miltefosine for the treatment of American cutaneous leishmaniasis. Am J Trop Med Hyg 83: 351–356. 20682881 10.4269/ajtmh.2010.10-0060 PMC2911184

[76] Miranda-Verastegui C, Tulliano G, Gyorkos TW, Calderon W, Rahme E, et al (2009) First-line therapy for human cutaneous leishmaniasis in Peru using the TLR7 agonist imiquimod in combination with pentavalent antimony. PLoS Negl Trop Dis 3: e491. 19636365 10.1371/journal.pntd.0000491 PMC2710502

[77] Soto J, Rea J, Balderrama M, Toledo J, Soto P, et al (2008) Efficacy of miltefosine for Bolivian cutaneous leishmaniasis. Am J Trop Med Hyg 78: 210–211. 18256415

[78] Soto J, Rea J, Valderrama M, Toledo J, Valda L, et al (2009) Efficacy of extended (six weeks) treatment with miltefosine for mucosal leishmaniasis in Bolivia. Am J Trop Med Hyg 81: 387–389. 19706901

[79] Sousa AQ, Frutuoso MS, Moraes EA, Pearson RD, Pompeu MM (2011) High-dose oral fluconazole therapy effective for cutaneous leishmaniasis due to Leishmania (Vianna) braziliensis. Clin Infect Dis 53: 693–695. 21890773 10.1093/cid/cir496

[80] Motta JO, Sampaio RN (2012) A pilot study comparing low-dose liposomal amphotericin B with N-methyl glucamine for the treatment of American cutaneous leishmaniasis. J Eur Acad Dermatol Venereol 26: 331–335. 21492255 10.1111/j.1468-3083.2011.04070.x

[81] Lopez-Jaramillo P, Rincon MY, Garcia RG, Silva SY, Smith E, et al (2010) A controlled, randomized-blinded clinical trial to assess the efficacy of a nitric oxide releasing patch in the treatment of cutaneous leishmaniasis by Leishmania (V.) panamensis. Am J Trop Med Hyg 83: 97–101. 20595484 10.4269/ajtmh.2010.09-0287 PMC2912582

[82] Llanos-Cuentas A, Calderon W, Cruz M, Ashman JA, Alves FP, et al (2010) A clinical trial to evaluate the safety and immunogenicity of the LEISH-F1+MPL-SE vaccine when used in combination with sodium stibogluconate for the treatment of mucosal leishmaniasis. Vaccine 28: 7427–7435. 20851080 10.1016/j.vaccine.2010.08.092

[83] Nascimento E, Fernandes DF, Vieira EP, Campos-Neto A, Ashman JA, et al (2010) A clinical trial to evaluate the safety and immunogenicity of the LEISH-F1+MPL-SE vaccine when used in combination with meglumine antimoniate for the treatment of cutaneous leishmaniasis. Vaccine 28: 6581–6587. 20688040 10.1016/j.vaccine.2010.07.063

[84] Garcia RG, Rincon Acelas M, Silva SY, Lopez M, Velez ID, et al. Double-blind, randomised controlled trial, to evaluate the effectiveness of a controlled nitric oxide releasing patch versus meglumine antimoniate in the treatment of cutaneous leishmaniasis; 2009; Helsinki Finland. 10.1186/1745-6215-7-14 PMC1524981 16700912

[85] Almeida OLS, Santos JB (2011) Advances in the treatment of cutaneous leishmaniasis in the New World in the last ten years: a systematic review. An Bras Dermatol 86: 497–506. 21738967 10.1590/s0365-05962011000300012

[86] Oliveira LF, Schubach AO, Martins MM, Passos SL, Oliveira RV, et al (2011) Systematic review of the adverse effects of cutaneous leishmaniasis treatment in the New World. Acta Trop 118: 87–96. 21420925 10.1016/j.actatropica.2011.02.007

[87] Baccan GC, Oliveira F, Sousa AD, Cerqueira NA, Costa JM, et al (2011) Hormone levels are associated with clinical markers and cytokine levels in human localized cutaneous leishmaniasis. Brain Behav Immun 25: 548–554. 21182928 10.1016/j.bbi.2010.12.009

[88] Romero GA, Guerra MV, Paes MG, Macedo VO (2001) Comparison of cutaneous leishmaniasis due to Leishmania (Viannia) braziliensis and L. (V.) guyanensis in Brazil: therapeutic response to meglumine antimoniate. Am J Trop Med Hyg 65: 456–465. 11716098 10.4269/ajtmh.2001.65.456

[89] Llanos-Cuentas A, Tulliano G, Araujo-Castillo R, Miranda-Verastegui C, Santamaria-Castrellon G, et al (2008) Clinical and parasite species risk factors for pentavalent antimonial treatment failure in cutaneous leishmaniasis in Peru. Clin Infect Dis 46: 223–231. 18171254 10.1086/524042

[90] Seiffert K (2011) Structures, targets and recent approaches in antileishmanial drug discovery and development. The open medicinal chemistry journal 5: 31–39. 21629509 10.2174/1874104501105010031 PMC3103891

[91] Blum J, Lockwood DNJ, Visser L, Harms G, Bailey MS, et al (2012) Local or systemic treatment for New World Cutaneous Leishmaniasis? Re-evaluating the evidence for the risk of mucosal leishmaniasis. International Health 4: 153–163. 24029394 10.1016/j.inhe.2012.06.004

[92] González U, Pinart M, Reveiz L, Rengifo-Pardo M, Tweed J, et al (2010) Designing and reporting clinical trials on treatments for cutaneous leishmaniasis. Clin Infect Dis 51: 409–19. 20624067 10.1086/655134

[93] da Graca GC, Volpini AC, Romero GA, de Oliveira Neto MP, Hueb M, et al (2012) Development and validation of PCR-based assays for diagnosis of American cutaneous leishmaniasis and identification of the parasite species. Mem Inst Oswaldo Cruz 107: 664–674. 22850958 10.1590/s0074-02762012000500014

[94] Adaui V, Maes I, Huyse T, Van den Broeck F, Talledo M, et al (2011) Multilocus genotyping reveals a polyphyletic pattern among naturally antimony-resistant Leishmania braziliensis isolates from Peru. Infect Genet Evol 11: 1873–1880. 21871584 10.1016/j.meegid.2011.08.008

[95] Torres DC, Adaui V, Ribeiro-Alves M, Romero GA, Arevalo J, et al (2010) Targeted gene expression profiling in Leishmania braziliensis and Leishmania guyanensis parasites isolated from Brazilian patients with different antimonial treatment outcomes. Infect Genet Evol 10: 727–733. 20478409 10.1016/j.meegid.2010.05.006

[96] Faria DR, Souza PE, Duraes FV, Carvalho EM, Gollob KJ, et al (2009) Recruitment of CD8(+) T cells expressing granzyme A is associated with lesion progression in human cutaneous leishmaniasis. Parasite Immunol 31: 432–439. 19646207 10.1111/j.1365-3024.2009.01125.x PMC2764276

[97] Cruz A, Rainey PM, Herwaldt BL, Stagni G, Palacios R, et al (2007) Pharmacokinetics of antimony in children treated for leishmaniasis with meglumine antimoniate. J Infect Dis 195: 602–608. 17230422 10.1086/510860

