# Supplementary material for: Interventions for American Cutaneous and Mucocutaneous Leishmaniasis: A Systematic Review Update
Source: PLoS One. 2013 Apr 29;8(4):e61843. doi: 10.1371/journal.pone.0061843 (PMC3639260; doi:10.1371/journal.pone.0061843)
Supplement: Supporing Information S1 — Search strategies. (DOC) [file pone.0061843.s001.doc]

**Supporting information S1. Search strategies**

**PubMed (August 29, 2012): 787**

(leishmaniasis [mh] OR leishmania [mh] OR leishma* [tw]) AND (randomized controlled trial[pt] OR controlled clinical trial[pt] OR randomized[tiab] OR placebo[tiab] OR drug therapy[sh] OR randomly[tiab] OR trial[tiab] OR groups[tiab] OR "treatment studies"[tiab] OR "therapy studies"[tiab]) NOT (animals[mh] NOT (humans[mh] AND animals[mh]))

**Lilacs (August 29, 2012): 541**

Tw estud$ OR Tw clin$ OR AB grupo$ OR CT COMPARATIVE STUDY OR Tw placebo$ OR Tw random$ OR Ti compara$ OR Ti tratamiento OR Tw control$ OR MH /dt [Words] and MH leishmaniasis OR MH leishmania OR Tw leishmania$ [Words]

**Embase (1980 to July 31, 2011): 155**

#21 #20 AND #19

#20 #1 OR #2 OR #3

#19 #13 NOT #18

#18 #14 NOT #15

#15 'human'/syn AND [embase]/lim

#14 'animals'/exp AND [embase]/lim

#13 #4 OR #5 OR #6 OR #7 OR #8 OR #9 OR #10 OR #11 OR #12

#12 groups:ab AND [embase]/lim

#11 trial:ab AND [embase]/lim

#10 randomly:ab AND [embase]/lim

#9 'drug therapy'/syn AND [embase]/lim

#8 placebo:ab AND [embase]/lim

#6 'controlled clinical trial'/exp AND [embase]/lim

#5 'randomized controlled trial'/exp AND [embase]/lim

#4 randomized:ab AND [embase]/lim

#3 leishmania* AND [embase]/lim

#2 'leishmania'/exp AND [embase]/lim

#1 'leishmaniasis'/exp AND [embase]/lim

**Scirus (Limits: medicine; July 31, 2011):**

(title:leishmaniasis OR title:leishmania) AND (randomized OR randomised OR random) AND trial

**Cochrane Central Register of Controlled Trials (7; 2012); 382**

Leishmania OR leishmaniasis (Mesh and text word)

**International Clinical Trial Platform Registry (July 31, 2011)**

Leishmania OR leishmaniasis
